# Evaluation of retinal microvascular perfusion in hereditary angioedema: a case-control study

**DOI:** 10.1186/s13023-019-1263-6

**Published:** 2020-01-17

**Authors:** Paola Triggianese, Massimo Cesareo, Maria Domenica Guarino, Paola Conigliaro, Maria Sole Chimenti, Francesca Cedola, Caterina Mazzeo, Carlo Nucci, Roberto Perricone

**Affiliations:** 1 Rheumatology, Allergology and Clinical Immunology, Department of “Medicina dei Sistemi”, University of Rome Tor Vergata, Rome, Italy; 2 Ophthalmology Unit, Department of Experimental Medicine and Surgery, University of Rome Tor Vergata, Rome, Italy

**Keywords:** Capillaries, Complement system, Hereditary angioedema, Optical coherence tomography angiography, Retina

## Abstract

Evidence supports that hereditary angioedema (HAE) may be considered as a paroxysmal permeability disorder with defective but self-limiting endothelial barrier dysfunction. A potential subclinical abnormal vascular permeability at retinal capillaries could induce damage resulting in retinopathy. We aimed at exploring for the first time the presence of microangiopathy at retinal level from a highly selective cohort of patients with HAE due to C1 esterase inhibitor protein (C1INH) deficiency (type I). We conducted a pilot, prospective, case-control study including 20 type I HAE patients and 20 age−/sex-matched healthy controls (HC). All participants underwent standard ophthalmological examination including visual fields. Superficial and deep capillary plexi in the retina were analyzed by using new optical coherence tomography angiography (OCT-A). A total of 40 eyes from 20 HAE patients and 20 eyes from HC were evaluated. Perimetric indices of visual field were slightly worse in HAE than in controls. OCT-angiograms documented in HAE patients a lower retinal capillary density in both superficial and deep scans and a higher retinal thickness compared to healthy eyes. Our findings firstly documented subclinical abnormalities in retinal microvascular network in type I HAE patients that might be associated with early subtle functional changes. This preliminary evidence supports the hypothesis of a recurrent endothelial barrier failure at retinal level in HAE patients potentially resulting in chronic damage.

## Background

Hereditary angioedema (HAE) due to C1 esterase inhibitor protein (C1INH) deficiency (type I HAE) is a rare disorder that is characterized by widely variable and potentially fatal attacks of subcutaneous and submucosal edema [1, 2]. Evidence supports that the contact activation resulting in the increase of vascular permeability during HAE attack is both a systemic and a local process at the affected tissues [3]. Accordingly, HAE may be considered as a paroxysmal permeability disorder with defective but self-limiting endothelial barrier dysfunction induced by a long list of mediators [4]. Angiopoietins and Vascular Endothelial Growth Factors (VEGFs) have been implicated in endothelial barrier failure in HAE as observed in diseases associated with higher vascular permeability (e.g., systemic capillary leak syndrome) [4–6]. As known, kinins play a primary role in the development of retinopathy through inflammatory mechanisms and enhanced vascular permeability that can be explained by an increase in bradykinin levels [7, 8]. In this context, the analysis of increased vascular permeability in retina might be a reasonable tool to explore vasoregulatory aspects of HAE due to C1INH deficiency. Optical Coherent Tomography Angiography (OCT-A) is a non-invasive imaging technique that is able to visualize the retinal microvasculature detecting blood flow without intravenous dye injection [9, 10]_._

We conducted a pilot, prospective, case-control study in order to explore for the first time the presence of a subclinical microangiopathy at retinal level from a highly selective cohort of type I HAE patients by using OCT-A.

## Patients and methods

We enrolled 20 type I HAE patients referring to our tertiary center for HAE (“Policlinico Tor Vergata”, Rome, Italy) during a 3-month period (May–July 2018). Inclusion criteria were: 1) diagnosis of type I HAE [1, 2]; 2) age ≥ 18/≤ 75 years old; 3) intraocular pressure (IOP) < 21 mmHg; 4) best-corrected visual acuity (BCVA) ≥ 0.5 LogMAR; 5) spherical equivalent refractive error between − 6.0/+ 4.0 diopters; 6) open anterior chamber angle on slit-lamp examination [11, 12]. Exclusion criteria were: 1) established primary ocular diseases; 2) systemic disorders including hypertension and/or treatments that may affect retinal function; 3) pregnancy or lactation; 4) neoplasia [11, 12]. The control group consisted of 20 (normal) subjects of the same age range and sex as the HAE patients recruited at the Ophthalmology Clinic (“Policlinico Tor Vergata”, Rome, Italy). Both eyes of each control were evaluated, however only one eye was randomly chosen for statistical analysis. The same exclusion criteria were applied to the control group.

The study described has been carried out in accordance with The Code of Ethics of the World Medical Association (Declaration of Helsinki) for experiments involving humans (updated 2008). Written informed consent was obtained from patients and controls and the study was approved by the local ethic (“Policlinico Tor Vergata”, Rome, Italy). At the enrollment, clinical records of 20 patients were registered from all HAE patients including family history, HAE disease duration, sites and severity of attacks, time interval between the last attack and the visit, concomitant disorders, on-demand and prophylactic HAE treatment. From each patient, laboratory assays were performed on the same day of the ophthalmological examination at the laboratory of the “Policlinico Tor Vergata” (Rome, Italy) and included measurements of serum C3, C4, antigenic and functional C1INH, and C1q levels (see Additional file 1) [2, 13]. Systolic and diastolic blood pressure was measured, and the derived mean arterial blood pressure (MABP) was assessed from all the subjects in the study [14, 15].

All subjects underwent a standard ophthalmological examination, including best-corrected visual acuity (BCVA), intraocular pressure (IOP), and visual field test (VF).

BCVA was measured using a standard LogMAR eye chart according to the Early Treatment of Diabetic Retinopathy Study (ETDRS) protocol: low vision is defined as a best-corrected visual acuity worse than 0.5 LogMAR [16]. IOP was measured by using Goldmann applanation tonometry by estimating the force (mmHg) required to applanate a constant area of the cornea [17]. Visual field defects were assessed using Humphrey Field Analyzer (HFA; model 750, Zeiss Humphrey Systems, Dublin, CA, USA), using the SITA-Standard program 30–2. Mean deviation (MD), pattern standard deviation (PSD), and visual field indexes (VFI) were calculated separately for each eye and compared between groups [18]. All the eyes were examined by means of a 6 × 6 mm optical coherence tomography angiography (OCT-A) scans (Optovue XR Avanti, Fremont, CA) [9]. Split-spectrum amplitude-decorrelation angiography generated OCT-angiograms of both superficial and deep retinal capillary plexi referred to the whole image, foveal, and parafoveal zone. The default en face display slab definition was 3 μm below the inner limiting membrane (ILM) to 15 μm below the inner plexiform layer (IPL) for the superficial capillary plexus, and 15 μm to 70 μm below the IPL for the deep capillary plexus [19, 20]. The segmentation software automatically detected the boundaries of the retinal layers from the structural OCT cross-sectional images. Retinal layer segmentation was checked for artifacts by the same experienced operator. Only images with quality > 8 have been considered for the study. It was not necessary to perform manual corrections; the Optovue XR Avanti (Fremont, CA) has active motion-tracking capability to minimize motion artifacts [21]. The OCT-A measurements were performed at the same time of the day (specifically, 3:00–5:00 pm) in both patients and controls [14, 22–24]. Thicknesses at the whole image, foveal, and parafoveal zone were measured by OCT [9, 10, 25].

Normally distributed variables were expressed by mean and standard deviation (SD). Continuous variables were compared using the parametric unpaired T test or the nonparametric Mann–Whitney U test when appropriate. The significance of any correlation was determined by Pearson correlation test. A sample size calculation was performed: the power analysis reached a value of 99% by using 2 groups of 20 subjects each one (20 cases and 20 controls) with an error α = 0.05 and an error β = 0.1. *P* values < 0.05 were considered significant (GraphPad Prism version 7; software for Power Analysis and Sample Size: NCSS 12 and PASS 16).

## Results and discussion

A total of 40 eyes of 20 type I HAE patients (50% female) from 12 independent families were included: the confirmation of the inheritance was based mainly on the family history, and genetic testing was conducted on 12 cases [26]. Demographic and clinical data from the study population were described in Table 1.

**Table 1:** Data from the study population

	HAE (*n* = 20)	HC (*n* = 20)
Age (years)	41.3 ± 13.5	45 ± 13
Female sex (N/%)	10/50	11/55
HAE disease duration (years)	27.4 ± 14	N.A.
Number of attacks^a^	8 ± 7.6	N.A.
Attack-free period (days)^b^	59 ± 71	N.A.
Danazol prohylaxis (N/%)	4/20	N.A.
C4 (mg/dl)	9.5 ± 4.5	N.A.
C1q (mg/l)	146 ± 20.3	N.A.
C1INH (mg/dl)	7 ± 3.4	N.A.
C1INH (%)	27.6 ± 12.2	N.A.
MABP (mmHg)	89 ± 10.4	88 ± 8
BCVA (LogMAR)	0.01 ± 0.1 (R); 0.01 ± 0.1 (L)	0.013 ± 0.03
IOP (mmHg)	16.5 ± 3 (R); 16.7 ± 2.9 (L)	16 ± 3
MD (median, dB)	−1.9 ***(R); − 2 ***(L)	0.3
PSD (median, dB)	2.16 **(R); 1.9 *(L)	1.6
VFI (range %)	92–98	98–100

*HAE* hereditary angioedema, *HC* healthy controls, *C1INH* C1 inhibitor, *MABP* mean arterial blood pressure, *BCVA* best corrected visual acuity, *IOP* intraocular pressure, *MD* mean deviation, *PSD* Pattern Standard Deviation, *VFI* visual field index, *R* right eyes, *L* left eyes. Continuous variables were shown using mean and standard deviation (SD) while categorical variables with absolute frequencies and percentages. Values from patients were compared with controls using the parametric unpaired T test or the nonparametric Mann–Whitney U test when appropriate and *P* values < 0.05 were considered significant (*p < 0.05, **p < 0.01, ***p < 0.001, with the respect to control eyes). ^a^number of HAE attacks in the last 12 months; ^b^number of days from the last acute attack to the time of the visit

HAE patients showed a median MD value lower than the controls (Table 1). In addition, the median PSD from HAE patients was higher than the controls (Table 1). VFI were similar in HAE patients and controls (Table 1).

**Fig. 1:**
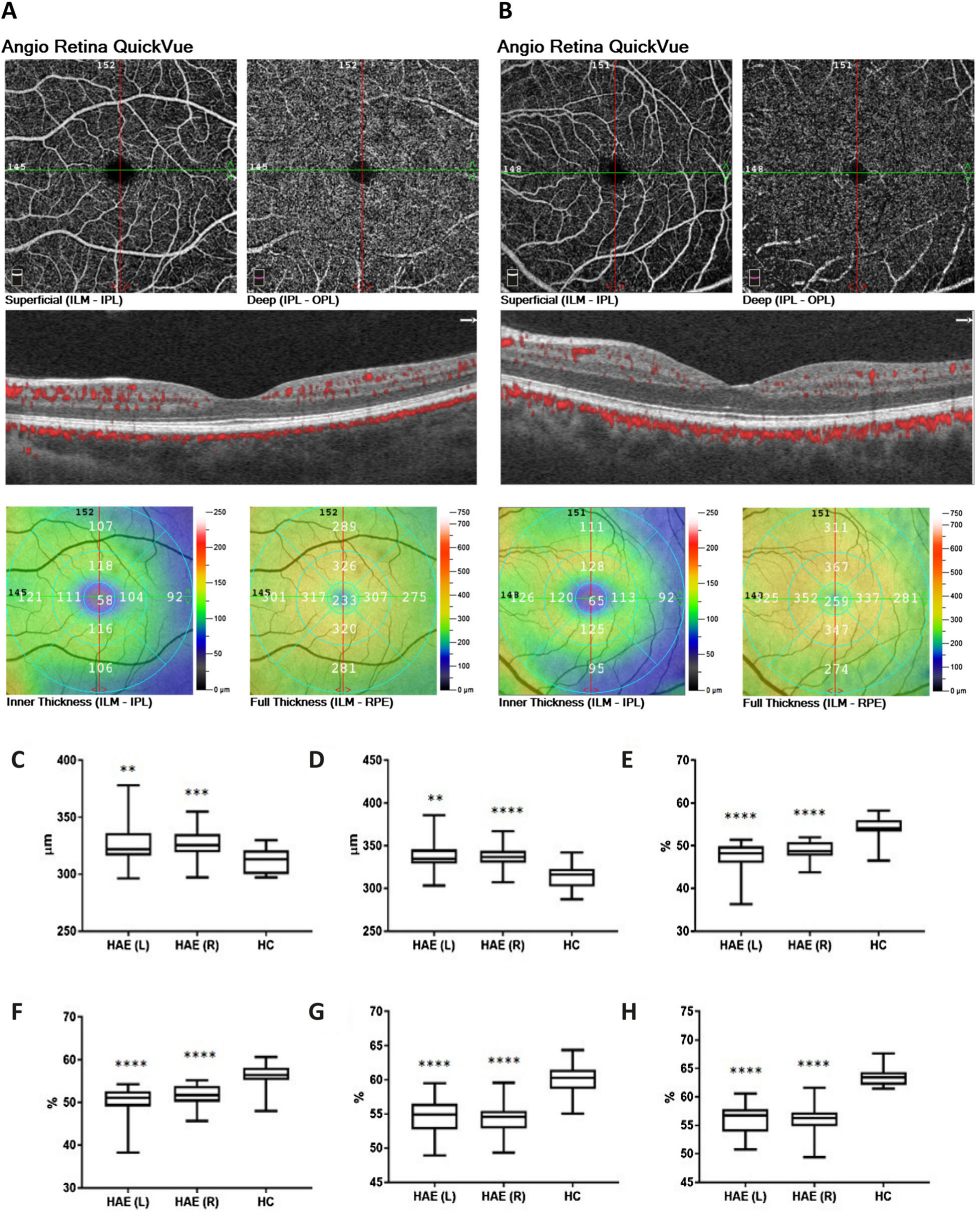
Retinal imaging by optical coherence tomography angiography. Optical coherence tomography angiography (OCT-A) generated en face 6 × 6-mm angiograms of superficial and deep retinal capillary plexi: representative scans from the left eye of a healthy control (HC) and a patient with type I hereditary angioedema (HAE) were reported in panels (**a** and **b**), respectively. Color-coded topographic maps described corresponding thicknesses with quantitative data. HAE patients showed higher retinal thickness at whole image scan (panel **c**, left *P* = 0.006; right *P* = 0.0008) and at the parafoveal area (panel **d**, left *P* = 0.006; right *P* < 0.0001) than controls. Superficial and deep retinal capillary density at whole image (panel **e** and **g**, respectively) and at the parafoveal area (panel **f** and **h**, respectively) was lower in HAE patient than in control with *P* < 0.0001 for all the comparisons between HAE and HC. Data are reported as box and whisker plots with median, lower as well as upper extreme. Significant differences were tested using the Mann–Whitney U test. *P* < 0.05 was considered statistically significant. (** *P* < 0.01, *** *P* < 0.001, **** *P* < 0.0001 with respect to control eyes). L, left eyes; R, right eyes; ILM, inner limiting membrane; IPL, inner plexiform layer; OPL, outer plexiform layer; RPE, retinal pigment epithelium

Retinal microvascular perfusion was analyzed at both superficial and deep capillary plexi by using OCT-A. Representative scans of the 6 × 6-mm angiograms by OCT-A from a HC and a HAE patient are depicted in Fig. 1. Retinal thickness measured by OCT was greater in HAE patients than that in controls at whole image scans (right *P* = 0.0008; left *P* = 0.006) and at the parafoveal area (left *P* = 0.006; right *P* < 0.0001) (Fig. 1). Compared to controls, HAE patients showed a lower superficial and deep capillary density at the whole image scan and parafoveal area (*P* < 0.0001 for each comparison) (Fig. 1; see Additional file 1: Table S1).

No significant correlations occurred between OCT-A findings and C4, C3, C1INH antigen and functional levels, and C1q. Retinal microvascular parameters did not correlate with age of patients at the visit, HAE disease duration, number of attacks in the last 12 months to the visit as well as in accordance with the duration of the interval between the last acute attacks and the study (Table 1).

Summarizing our findings, in HAE patients, we might assume that a subclinical edema formation increases the thickness of the parafoveal area and leads to a “relative” reduction of the vascular network by compressing the existing capillaries [27, 28]. The significant reduction in vessel density values that was detected in HAE cohort looks like the low vessel density documented in patients with papilledema and pseudopapilledema in whom hypothetically the apparent reduced density of vessels on OCT-A might be due to slow flow resulting from compression/stasis [28, 29]. In this view, in C1INH deficiency-HAE the endothelial barrier failure with enhanced vascular permeability can likely involve tissues and organs at the site of attacks [3]. However, this conceivable subclinical edema at retinal level may lead to a chronic damage resulting in early subtle functional changes like anomalies in perimetric indices documented in our cohort. Mild visual field defects documented in HAE patients may have been for a long time undetected because of insufficient eye examination. To date, there is a paucity of recommendations that reliably detect retinal involvement in HAE: specifically, only for HAE patients undergoing prophylactic antifibrinolytic treatment, annual ophthalmologic examinations are recommended [30].

As a remark, no significant correlations between OCT-A parameters and complement components levels occurred. Anyway, our data cannot exclude definitely the potential correlations between complement levels and retinal microvasculature because HAE patients have not been evaluated during an acute attack and both prophylactic and on-demand treatment likely affect circulating complement levels as well as the disease course [2]. Accordingly, no significant correlations occurred between OCT-A findings and number or severity of attacks in our cohort. Furthermore, when stratifying patients in accordance with danazol treatment, four patients were on long-term therapy at the time of the study while eight had a history of a previous danazol long-term prophylaxis (the mean danazol-free period at the enrollment was 7 ± 3 years). We did not register differences in the capillary density scans and in the retinal thickness by OCT-A between patients who have been on danazol (*n* = 12) and patients never taking danazol (*n* = 8). In addition, we did not register differences in the capillary density scans and in the retinal thickness by OCT-A between patients taking C1INH replacement therapy (*n* = 9) and patients who did not take it (*n* = 11) in the last 3 months.

Further investigations including a larger HAE cohort might lead to an improvement of the analysis by comparing among subgroups of patients (e.g., between patients currently taking androgens and having ever taken androgens, as well as patients experiencing treated vs. untreated attacks). In addition, future studies on structural analysis of the retina are certainly awaited [29, 31].

Our pilot proof-of-concept study for the first time provided evidence of retinal microcirculatory abnormalities in HAE patients: these early microvascular changes revealed by OCT-A could precede clinical neuro-retinopathies.

## Supplementary information

**Additional file 1. **Immunologic assessment. **Table S1.** Optical coherence tomography angiography findings from the study population. **Figure S1.** Representative 3 × 3-mm angiograms of retinal imaging on optical coherence tomography angiography.

## Data Availability

The datasets used and/or analysed during the current study are available from the corresponding author on reasonable request.

## Abbreviations

BCVA: Best corrected visual acuity
C1INH: C1 inhibitor
HAE: Hereditary angioedema
HC: Healthy controls
IOP: Intraocular pressure
MD: Mean deviation
OCT-A: Optical coherence tomography angiography
PSD: Pattern standard deviation
VEGF: Vascular Endothelial Growth Factors
VFI: Visual field index
