# Supplementary material for: Evaluation of retinal microvascular perfusion in hereditary angioedema: a case-control study
Source: Orphanet J Rare Dis. 2020 Jan 17;15:20. doi: 10.1186/s13023-019-1263-6 (PMC6969431; doi:10.1186/s13023-019-1263-6)

*Immunologic assessment*

Immunologic laboratory assays included C3, C4, antigenic and functional C1INH, and C1q levels. From all the patients in the study, measurements of serum C3 and C4 were obtained using nephelometric assays: normal values were 90-180 mg/dl and 10-40 mg/dl for C3 and C4, respectively. Serum C1q and C1INH antigen levels were measured with radial immunodiffusion while functional C1INH assessment was performed from citrate plasma with cromogenic method. Normal values were 15.4-35.1 mg/dl and 70-130% for antigenic and functional C1INH respectively, and 50-250 mg/l for C1q.

**Table S1. Optical coherence tomography angiography findings from the study population**

|  |  | Opthalmological scanning | HAE | | HC |
| --- | --- | --- | --- | --- | --- |
|  |  |  | right eyes  (n=20) | left eyes  (n=20) | control eyes  (n=20) |
| OCT-A | Capillary density (%) | Deep scans   - Fovea - Parafovea - Whole image   Superficial scans   - Fovea - Parafovea - Whole image | 36.4 ± 4.9  56 ± 2.4****  54.4 ± 2.3****  19.9 ± 3.9  51.7 ± 2.6****  48.7 ± 2.5**** | 35.8 ± 4.8  56 ± 2.5****  54.4 ± 2.7****  19 ± 3.6  50.3 ± 3.7****  47.5 ± 3.8**** | 31 ± 8.5  63 ± 2  60 ± 2  35.2 ± 5.9  56.3 ± 2.9  53.9 ± 3 |
|  | Thickness (µm) | - Fovea - Parafovea - Whole image | 263 ± 13.3  337.8 ± 13.3****  327 ± 12.6*** | 266.3 ± 17.4  336.5 ± 17.7***  325.7 ± 17.4** | 261 ± 20.3  317.8 ± 15.4  310 ± 11 |

HAE, hereditary angioedema; HC, healthy controls; OCT-A, Optical coherence tomography angiography. Continuous variables were shown using mean and standard deviation (SD). Values were compared using parametric unpaired T test or nonparametric Mann–Whitney U test when appropriate (*P* values <0.05 were considered significant (** P<0.01, *** P<0.001, **** P<0.0001 with respect to control eyes).

**Figure S1. Representative 3 × 3-mm angiograms of retinal imaging on optical coherence tomography angiography**

Optical coherence tomography angiography (OCT-A) generated en face 3× 3-mm angiograms of superficial retinal capillary plexus and deep retinal capillary plexus from the left eye of a healthy control (panel A) and a patients with type I hereditary angioedema (panel B). Color-coded topographic maps reported corresponding thicknesses with quantitative data.

ILM, internal limiting membrane; IPL, inner plexiform layer; OPL, outer plexiform layer; RPE, retinal pigment epithelium.


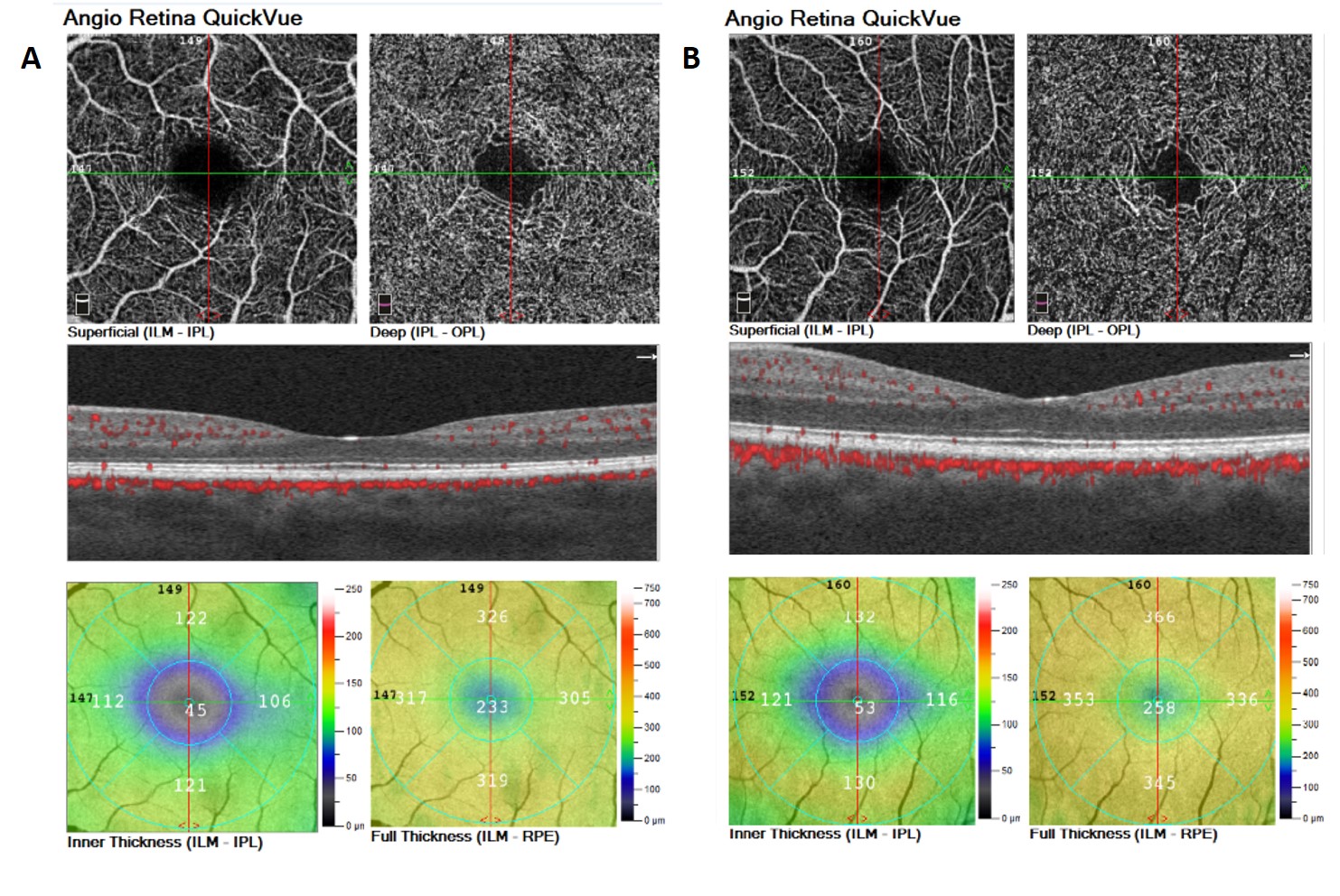

Supplement: Supplementary file 1 — Additional file 1. Immunologic assessment. Table S1. Optical coherence tomography angiography findings from the study population. Figure S1. Representative 3 × 3-mm angiograms of retinal imaging on optical coherence tomography angiography. [file 13023_2019_1263_MOESM1_ESM.doc]
